# FGFRL1 Promotes Ovarian Cancer Progression by Crosstalk with Hedgehog Signaling

**DOI:** 10.1155/2018/7438608

**Published:** 2018-02-20

**Authors:** Haiyan Tai, Zhiyong Wu, Su'an Sun, Zhigang Zhang, Congjian Xu

**Affiliations:** ^1^Obstetrics and Gynecology Hospital, Fudan University, Shanghai 200011, China; ^2^Department of Pathology, The First Hospital of Huai'an City, Nanjing Medical University, Huai'an, 223001 Jiangsu, China; ^3^State Key Laboratory of Oncogenes and Related Genes, Shanghai Cancer Institute, Shanghai Jiao Tong University, Shanghai 200240, China; ^4^Department of Obstetrics and Gynecology of Shanghai Medical School, Fudan University, Shanghai 200032, China; ^5^Shanghai Key Laboratory of Female Reproductive Endocrine Related Diseases, Shanghai 200011, China

## Abstract

Fibroblast growth factor receptor-like-1 (FGFRL1) has been identified as the fifth fibroblast growth factor receptor. So far, little is known about its biological functions, particularly in cancer development. Here, for the first time, we demonstrated the roles of FGFRL1 in ovarian carcinoma (OC). An array and existing databases were used to investigate the expression profile of FGFRL1 and the relationship between FGFRL1 expression and clinicopathological parameters. FGFRL1 was significantly upregulated in OC patients, and high FGFRL1 expression was correlated with poor prognosis. *In vitro* cell proliferation, apoptosis and migration assays, and *in vivo* subcutaneous xenograft tumor models were used to determine the role of FGFRL1. Loss of function of FGFRL1 significantly influenced cell proliferation, apoptosis, and migration of OC cells *in vitro* and tumor growth *in vivo*. Chromatin immunoprecipitation PCR analysis and microarray hybridization were performed to uncover the mechanism. FGFRL1 expression could be induced by hypoxia through hypoxia-inducible factor 1*α*, which directly binds to the promoter elements of FGFRL1. FGFRL1 promoted tumor progression by crosstalk with Hedgehog (Hh) signaling. Taken together, FGFRL1 is a potential predictor and plays an important role in tumor growth and Hh signaling which could serve as potential therapeutic targets for the treatment of OC.

## 1. Introduction

Ovarian carcinoma (OC) has the highest mortality rate among the malignancies of the female reproductive tract. In the United States, more than 22,000 women were diagnosed with OC and more than 14,000 women died in 2016 [1]. The poor survival rate is due primarily to the advanced stage of disease and widespread metastases at the time of diagnosis. Therefore, an insight into the understanding of the molecular mechanisms underlying the progression of OC and identifying new targets and strategies is pressing.

Fibroblast growth factor-like-1 (FGFRL1) is a member of the fibroblast growth factor receptor (FGFR) family [2–6]. Fibroblast growth factors (FGF) and their receptors are known for regulating numerous cellular processes. Activated FGFRs have become a promising potential target in many cancers, including ovarian carcinoma. FGFRL1 encoding the protein composed of a cytoplasmic His-rich motif without the intracellular tyrosine kinase domain. Owing to the fact, it was thought to be a decoy receptor and exert no or negative effect on cell proliferation in some studies [7, 8]. However, FGFRL1 is involved in multiple cellular functions. FGFRL1 was reported to take part in the progression of endometriosis [9] and induce cell-cell fusion in CHO cells [10]. Targeted deletion of FGFRL1 leads to severe kidney dysgenesis [11]. FGFRL1 was identified to enhance ERK1/2 signaling through association of SHP with the receptor's intracellular SH2-binding motif in beta-cells in the pancreas [12]. In high-grade serous ovarian tumor, FGFRL1 mRNA isoform was identified with tumor-specific expression [13]. FGFRL1 could accelerate tumor growth in different neoplastic diseases [14–16]. However, little is known about cellular functions of FGFRL1 in ovarian carcinoma.

Herein, we analyzed the expression pattern and clinical significance of FGFRL1 in ovarian cancer and tried to detect important pathways as well as key genes in order to understand the mechanism of FGFRL1 contributing to the development of ovarian cancer.

## 2. Material and Methods

### 2.1. Case Cohort

Human ovarian cancer tissue microarrays containing 90 cases of ovarian carcinoma and 10 cases of normal ovarian tissues were obtained from Obstetrics and Gynecology Hospital, Fudan University. The study was approved by the Research Ethics Committee of Obstetrics and Gynecology Hospital, Fudan University.

### 2.2. Cell Culture

Human ovarian cancer cell lines ES2, SKOV3, OVCAR8, Hey, and human immortalized ovarian epithelial cell line Moody were preserved at Shanghai Cancer Institute. All cells were cultured in indicated medium and supplemented with 10% fetal bovine serum (Invitrogen), 100ug/ml streptomycin, and 100 units/ml penicillin at 37°C with 5% CO_2_. Aiming to hypoxia treatment, OC cells were cultured in the incubator with 1% O_2_ gas mixture for the desired period of time. Prior to CHIP assay, Cobalt chloride (CoCl_2_) was used at the final concentration of 100 *μ*M in the OC cell culture media and incubated for 6 hours to induce hypoxia [17].

### 2.3. Immunohistochemistry

The tissue microarray (TMA) slide was prepared for immunohistochemistry (IHC) staining, and the primary antibody FGFRL1 (Rabbit polyclonal antibody, Novus) was used. The FGFRL1 staining intensity was scored as follows: negative, 0; weak, 1; moderate, 2; and strong, 3. Scoring of positive staining cells was conducted from 0–4: 0–5%, 0; 6–35%, 1; 36–70%, 2; and more than 70%, 3. The final score was designated using FGFRL1 staining intensity score × FGFRL1-positive cell score. The final score was determined as follows: a score of 0 or 1 was considered low expression, and a score of 2 or 3 was considered high expression. The results were evaluated by two experienced pathologists in a blinded manner.

### 2.4. Real-Time PCR

The total RNA was extracted from OC cells using RNAiso Plus (Takara, Tokyo, Japan). The reverse transcription was performed using a Prime-Script RT-PCR Kit (Takara, Tokyo, Japan). The qPCR was performed using ABI 7500 System (Applied Biosystems Inc. USA) with SYBR Green Master Mix (Takara, Japan). The data were analyzed to quantify the relative mRNA expression levels of genes. The primers used in this study are shown in Supplementary Table S2.

### 2.5. Western Blotting

OC cells were lysed in IP lysis buffer (Beyotime, Jiangsu, China) containing proteinase and phosphatase inhibitors (Selleck, TX, USA). Proteins were separated through SDS-PAGE and incubated overnight at 4°C using primary antibodies as follows: anti-FGFRL1 (Abcam, UK, ab112917, 1 : 100) and HIF1*α* (Abcam, UK, Ab16066, 1 : 1000). The bands were detected using ECL Western Blotting Detection Reagents (Millipore).

### 2.6. Short Interfering RNA-Based Gene Knockdown

OC Cells were transiently transfected using the Lipofectamine RNAiMAX reagent (Invitrogen), Opti-MEM reduced-serum medium (Invitrogen), and small interfering RNA (siRNA) oligonucleotides (Supplementary Table S1) for FGFRL1 silencing. After 48 hours, siRNA-treated cells were used in a subsequent experiment.

### 2.7. Cell Viability Assay

OC cells were transfected with FGFRL1 siRNA. After 48 hours, cell viability was measured using Cell Counting Kit-8 (CCK-8, Dojindo, Japan). The absorbance was detected at 450 nm using a microplate reader. The experiment was performed in triplicate and repeated triple.

### 2.8. Migration Assay

About 2 × 10^4^ cells in 200 *μ*L medium were seeded into the upper chambers (Corning, NY, USA) with an 8 *μ*m pore in 24-well plates. Medium containing 10% FBS was added to the lower chambers. After 16 hours, the cells which remained on the upper surface of the chambers were removed. The migrated cells were fixed and stained with crystal violet. The cells were counted in five random microscopic fields per well.

### 2.9. Wound Healing Assay

OC cells were seeded, and the wound was created by scraping with a pipette tip while cells were 90% confluent. After the debris was washed with PBS, photographs were taken to assess the ability of the cells to migrate into the wound area at 0 h and 24 h. Experiments were carried out in triplicate.

### 2.10. Apoptosis Assay

OC cells were cultured under serum deprivation overnight and detached with 0.25% trypsin without EDTA. Then cells were washed with 1 × PBS, stained with 50 *μ*g/ml propidium iodide and Annexin V-FITC (BD Pharmingen, USA) following the manufacturer's protocols. The percentage of Annexin V (+) and PI (−) cells was analyzed by flow cytometry.

### 2.11. Animal Experiments

Short hairpin RNA- (shRNA-) containing plasmids were packaged into lentivirus, and virus titers were determined. The sequence targeting FGFRL1 is as follows: sh: 5′-GTCGTGCTGGATGACATTAGC-3′. For *in vivo* tumor formation, 2 × 10^6^ sh-OVCAR8 cells were subcutaneously injected into one flank of each mouse. After 6 weeks, the mice were sacrificed, and the parameters were measured. Mice were manipulated and housed according to protocols approved by the East China Normal University Animal Care Commission.

### 2.12. Chromatin Immunoprecipitation (CHIP) Assay

CoCl_2_ was used to induce hypoxia in the hypoxia group. Cells were cross-linked with 1% formaldehyde, terminated by adding glycine (1.25 M), lysed, and fragmented. The extracts were incubated with the anti-HIF1*α* antibody or control IgG with rotation overnight at 4°C. After IP, the protein DNA cross-links were reversed. PCR was performed with the input DNA and the immunoprecipitates, and the products were separated by agarose gel electrophoresis. The primers used here are listed in Supplementary Table S2.

### 2.13. Dual-Luciferase Reporter Assay

FGFRL1 promoter-luciferase reporter plasmids were constructed in the pGL4 plasmid. A dual luciferase reporter assay (Promega, WI, USA) was performed following the manufacturer's instructions.

### 2.14. Microarray Hybridization

Si-control/ES2, Si-FGFRL1/ES2 cells, Si-control/OVCAR8, and Si-FGFRL1/OVCAR8 cells were homogenized in RNAiso Plus (Takara, Tokyo, Japan). The Affymetrix human genome U133 gene chip sets were performed by Shanghai Biotechnology Corporation. Transcript profiling was submitted to the National Center for Biotechnology Information's GEO database, and the repository URL and the data accession numbers are GSE 106549.

### 2.15. Statistical Analysis

The SPSS 19.0 software (IBM Corporation) was used for statistical analyses. The comparisons were taken using two-tailed paired Student's *t*-tests. The correlation was performed by a chi-square or Fisher's exact tests. Graphic representations were tested with GraphPad Prism software (San Diego, CA). For survival analysis, the Kaplan-Meier method was carried out and differences were analyzed by the log-rank test. Values of *p* < 0.05 were considered to be statistically significant.

## 3. Results

### 3.1. The Expression of FGFRL1 Is Upregulated in OC

**Figure 1:**
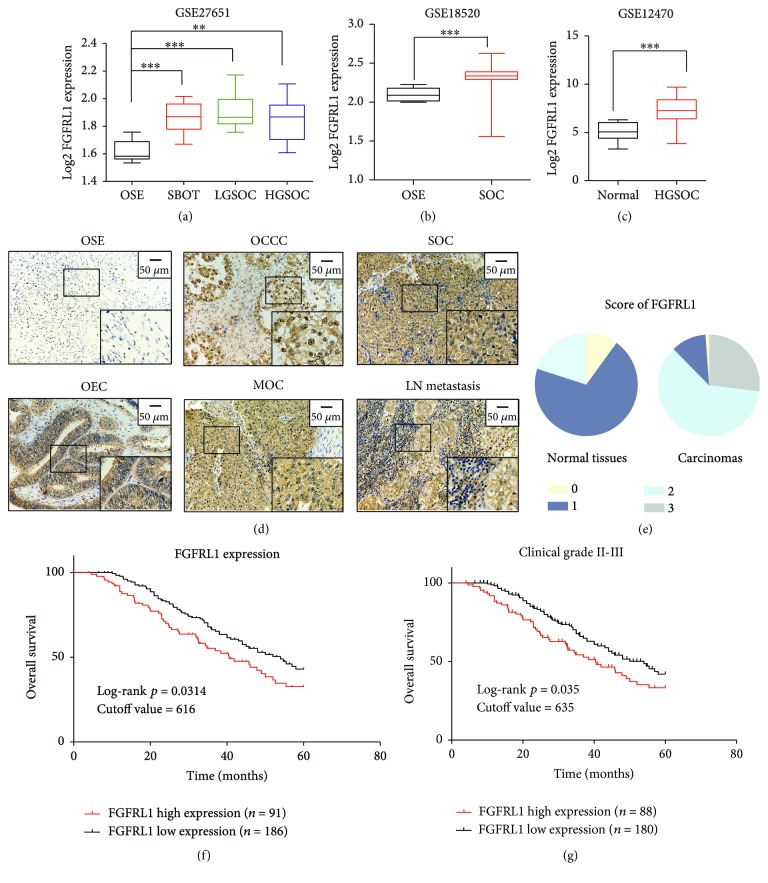
FGFRL1 expression is increased in OC. (a) The mRNA level of FGFRL1 is unregulated in SBOT, LGSOC, and HGSOC compared with OSE in the GSE27651 dataset. Comparison of mRNA expression levels of FGFRL1 in OSE and SOC tissues in the GSE18520 (b) and GSE12470 datasets (c). (d) The representative IHC staining of FGFRL1 in OC and noncancerous samples in the TMA. Scale bar, 50 *μ*m. (e) Score of the IHC staining in the TMA of OC. (f) Correlation between FGFRL1 expression and the patient overall survival was conducted in TCGA. (g) Comparisons of overall survival between the lower FGFRL1 expression group and the higher FGFRL1 expression group in patients with advanced grade (II-III). SBOT: serous borderline ovarian tumors; LGSOC: low-grade serous ovarian carcinomas; HGSOC: high-grade serous ovarian carcinomas; OSE: ovarian surface epithelia; SOC: serous ovarian carcinomas; OCCC: ovarian clear cell carcinoma; OEC: ovarian endometrioid adenocarcinoma; MOC: mucinous ovarian carcinoma; LN: lymphatic node. ^∗∗^ *p* < 0.01; ^∗∗∗^ *p* < 0.001.

By browsing databases, we found that FGFRL1 was upregulated in various tumors. We analyzed three independent microarray datasets from GEO datasets for FGFRL1 mRNA expression level. The results showed that FGFRL1 expression was significantly upregulated in serous borderline ovarian tumors (SBOT), low-grade serous ovarian carcinomas (LGSOC), and high-grade serous ovarian carcinomas (HGSOC) in comparison with ovarian surface epithelia (OSE) using GSE27651 (*n* = 49, *p* = 0.0007, *p* < 0.0001, and *p* = 0.0013; Figure 1(a)) [18]. FGFRL1 expression was also significantly higher in SOC tissues than OSE or normal tissues using GSE18520 (*n* = 63, *p* < 0.0001; Figure 1(b)) [19] and GSE12470 (*n* = 45, *p* < 0.0001; Figure 1(c)) [20]. Subsequently, to further address the protein change of FGFRL1 in OC, we performed IHC in a TMA of 90 OC samples and 10 noncancerous samples. The protein level of FGFRL1 was remarkably higher in OC tissues than that in normal tissue by IHC (Figures 1(d) and 1(e)).

### 3.2. Relationship between FGFRL1 Expression and Clinical Parameters of OC

To evaluate the clinical significance of FGFRL1 expression in OC, we assessed the relationship between FGFRL1 protein expression and clinical parameters (Supplementary Table S3). The results showed that FGFRL1 expression was significantly associated with histological grading.

### 3.3. High FGFRL1 Expression Predicts Poor Prognosis in OC

The correlation between FGFRL1 expression and clinical follow-up information was analyzed in order to evaluate the prognostic significance of FGFRL1 in OC patients. We evaluated the prognostic value of FGFRL1 at mRNA level using a Kaplan-Meier plotter tool with a total of 1648 cases enrolled from TCGA (the Cancer Genome Atlas) and multiple GEO datasets. As shown in Figure 1(f), patients with higher FGFRL1 level had significantly shorter survival time than those with a lower FGFRL1 level. Furthermore, the relationship between FGFRL1 expression and the overall survival in OC patients with advanced grade (II-III) was evaluated. The overall survival was shorter in advanced patients with high FGFRL1 expression (Figure 1(g)).

### 3.4. FGFRL1 Gene Is Infrequently Amplified and Could Be Induced by Hypoxia via Hypoxia-Inducible Factor 1*α* (HIF1*α*) in OC

**Figure 2:**
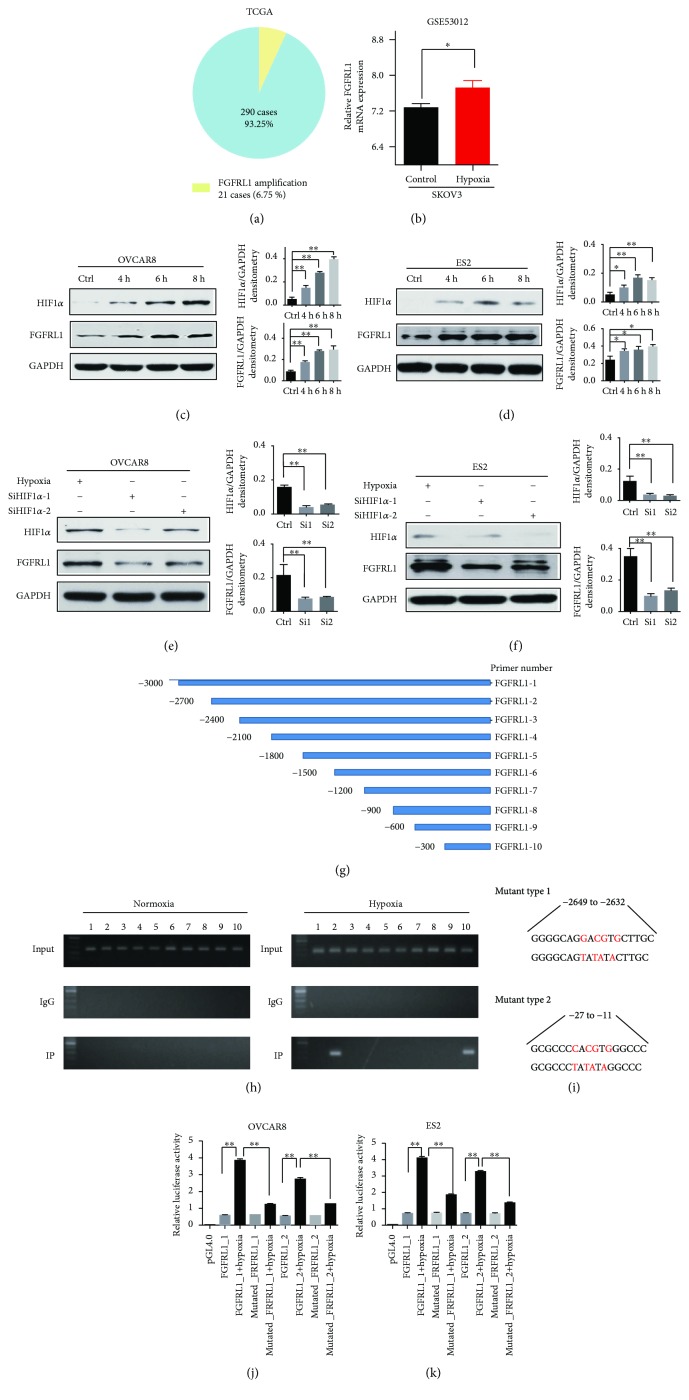
FGFRL1 could be induced by hypoxia via HIF1*α* in OC. (a) FGFRL1 DNA copy number amplification of OC in TCGA. (b) The mRNA expression level of FGFRL1 was significantly induced after hypoxia treatment in SKOV3 cells in the GSE53012 dataset. (c, d) The protein levels of FGFRL1 and HIF1*α* stimulated by hypoxia at indicated time intervals in OC cells. (e, f) The protein levels of FGFRL1 and HIF1*α* under HIF1*α* siRNA interference and hypoxia condition for 6 hours in OC cells. (g) 10 pairs of primers were constructed according to the promoter of FGFRL1. (h) A ChIP assay was performed to confirm the potential HIF1*α* binding site in the FGFRL1 promoter region. (i) Two putative HIF1*α*-binding sites in the FGFRL1 promoter located at −2649 to −2632 and −27 to −11 (mutation site: red). (j, k) A luciferase reporter assay was performed using OC cells after transfecting the wild-type plasmids and mutated plasmids. The data shown are the mean ± SD. ^∗^ *p* < 0.05; ^∗∗^ *p* < 0.01.

In order to identify whether gene amplification contributes to higher FGFRL1 expression in OC, our analysis of cancer genomics in TCGA revealed that gene amplification was infrequent in FGFRL1 gene and only 21 of 301 samples (6.75%; Figure 2(a)). The evidence is insufficient to explain the upregulation of FGFRL1 expression in OC.

Thus, we need more evidence to explain the upregulation of FGFRL1 expression. Hypoxia microenvironments are often found in many solid tumors including prostate cancer, brain tumor, and ovarian carcinomas [21–23]. Hypoxia treatments (2% O_2_) were performed in OC cells. The existing dataset showed that FGFRL1 expression was significantly induced after hypoxia treatment in SKOV3 cells in the GSE53012 dataset (Figure 2(b)). Our results revealed that the expression of FGFRL1 was induced by hypoxia treatment in OC cells at indicated time intervals (Figures 2(c) and 2(d)).

We tried to identify transcription factor responsible for hypoxia-induced upregulation of FGFRL1 expression. It has been reported that hypoxia-inducible factor 1 (HIF1) is a key molecule to help the hypoxia cells to compensate the hypoxia at the molecular level. Under hypoxic conditions, HIF-1*α* subunit can be stabilized and accumulated. Our results also suggest that hypoxia can induce the expression of HIF1*α* in OC cells.

To identify the role of HIF1*α*, we first examined the FGFRL1 expression level after HIF1*α* silencing with siRNAs under hypoxia condition for 6 hours. HIF1*α* knockdown reduced FGFRL1 expression in OC cells (Figures 2(e) and 2(f)). These findings suggest that FGFRL1 is the downstream target of HIF1*α* in OC cells. We hypothesized that HIF1*α* directly regulated FGFRL1 by transcription of the FGFRL1 promoter and conducted 10 pairs of primers according to the promoter of FGFRL1 (Figure 2(g)). We mimicked chemical hypoxia condition using CoCl_2_ in OC cell for 6 hours. CHIP PCR was performed in OC cell and indicated that HIF1*α* was directly bound to the second and tenth region in the FGFRL1 promoter under the hypoxia condition (Figure 2(h)). Two putative HIF1*α*-binding sites in the FGFRL1 promoter located at −2649 to −2632 and −27 to −11 were identified (Figure 2(i)). We constructed the wild-type and mutant FGFRL1 promoter luciferase reporters and used them in reporter assays. Reporter assays further confirmed that the transcriptional activity of FGFRL1 was significantly induced by HIF1*α* under hypoxia condition and was decreased by transduction of a mutant FGFRL1 promoter luciferase reporter (Figures 2(j) and 2(k)). Collectively, our findings supported that HIF1*α* could transcriptionally activate FGFRL1 by binding to two predicted sites in the FGFRL1 promoter.

### 3.5. FGFRL1 Affects OC Cell Proliferation, Apoptosis, and Cell Migration In Vitro

**Figure 3:**
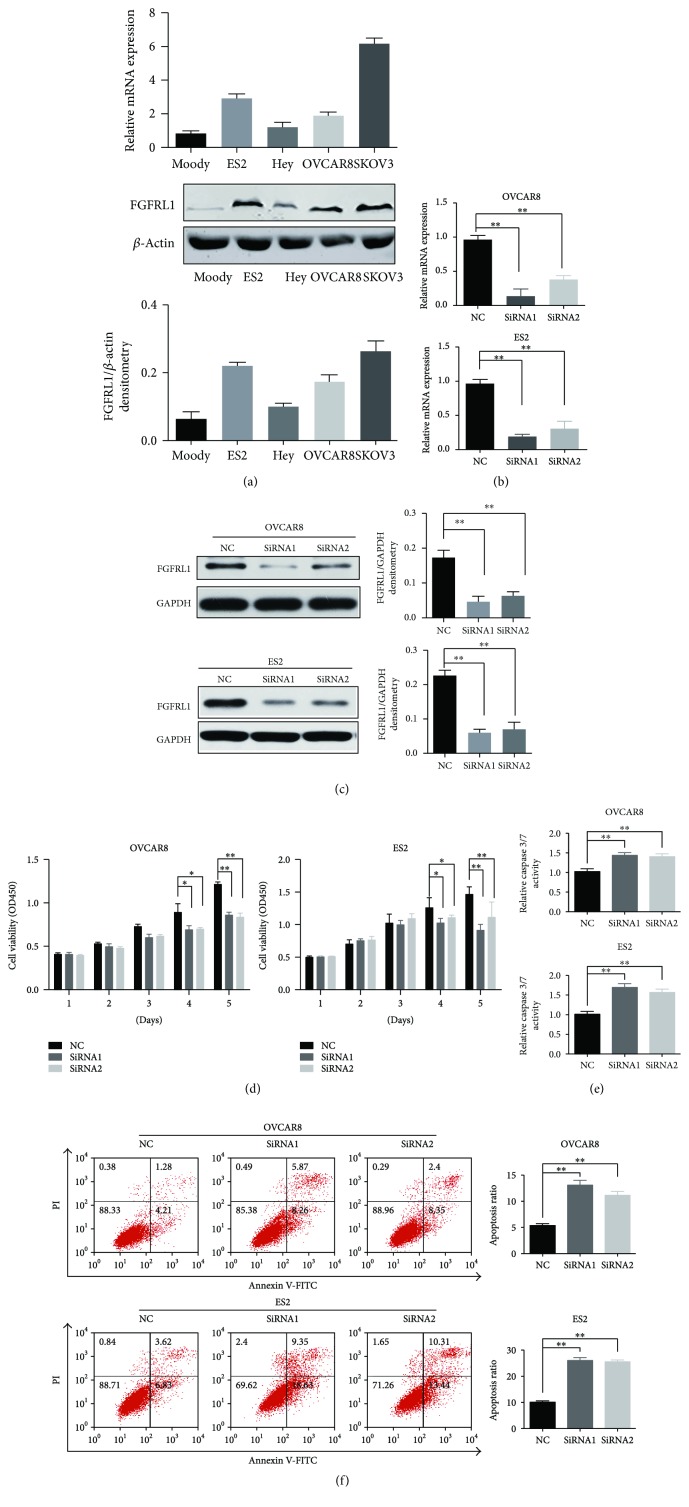
Silencing of FGFRL1 suppresses OC cell proliferation and promotes cell apoptosis. (a) The mRNA and protein expression levels of FGFRL1 were assessed in OC cell lines as well as a nonmalignant ovarian cell line Moody. (b) The qRT-PCR analysis of the FGFRL1 expression after FGFRL1 siRNA interference. (c) Successful FGFRL1 silencing was confirmed by Western blotting. (d) The effect of FGFRL1 on cell proliferation was determined by CCK8 assay. Knockdown of FGFRL1 promoted apoptosis as revealed by caspase-3/7 activity (e) and flow cytometry (f). ^∗^ *p* < 0.05; ^∗∗^ *p* < 0.01.

**Figure 4:**
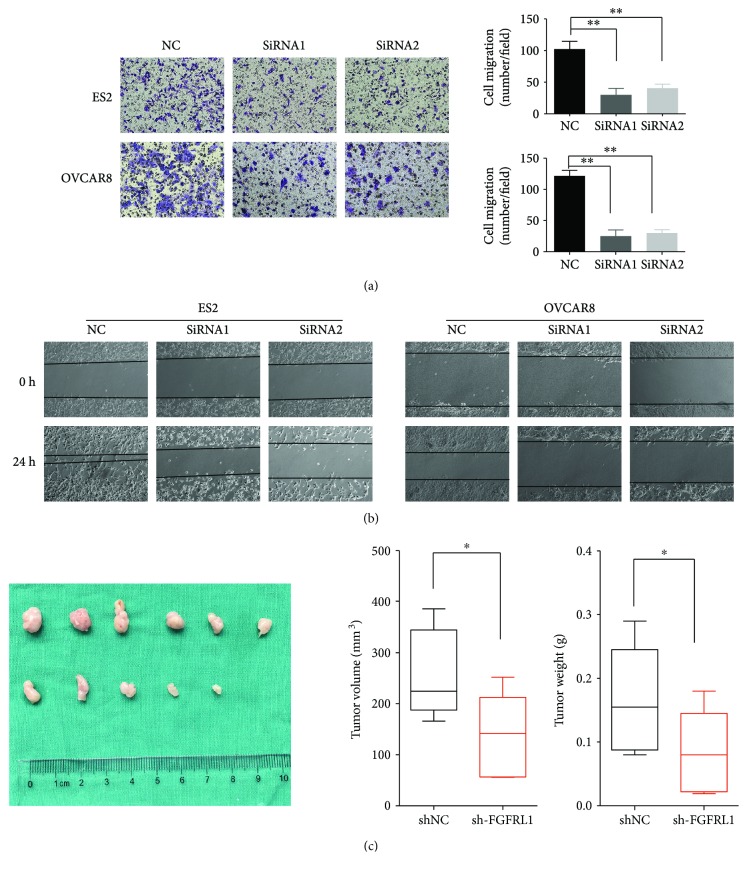
Silencing of FGFRL1 suppresses ovarian cancer migration *in vitro* and tumor growth *in vivo*. (a) Representative migration images of FGFRL1-silenced and control cells. Original magnification: 200x. Quantification of cells on the lower surface of the membrane was performed. Data are the means ± SD. (b) Representative wound healing images of FGFRL1-silenced and control cells at 0 and 24 h, respectively. The black line outlined the cell boundary. Quantification of wound healing rates was analyzed. Data are the means ± SD. (c) Mice in the sh-FGFRL1 group showed relatively larger tumors compared with those in the control group. ^∗^ *p* < 0.05; ^∗∗^ *p* < 0.01.

Based on FGFRL1 high expression with poor clinical prognosis, we further investigated the biological cellular functions of FGFRL1 in OC cells. Consistent with the findings in OC tissues, FGFRL1 expression was higher in OC cells than in a nonmalignant immortalized ovarian cell line Moody at protein level (Figure 3(a)). The siRNA-mediated loss of function for FGFRL1 resulted in >75% reduction in FGFRL1 expression in OC cells by RT-PCR and Western blot (Figures 3(b) and 3(c)). CCK-8 assay showed that knockdown of FGFRL1 significantly inhibited cell proliferation in OC cells (Figure 3(d)). The effect of FGFRL1 on cell apoptosis was also investigated by flow cytometric analysis. The silencing of FGFRL1 increased the apoptosis rate of OC cells (Figure 3(f)). Consistent with this, caspase-3/7 activity in OC cells was significantly increased by silencing of FGFRL1 (Figure 3(e)). The transwell model and wound healing assays were used to analyze the cell migration of OC cells after knockdown of FGFRL1 (Figures 4(a) and 4(b)).

### 3.6. FGFRL1 Suppresses Xenograft Tumor Growth In Vivo

FGFRL1 expression was reduced stably using shRNAs. To investigate the effect of FGFRL1 *in vivo*, OVCAR8 cells were transplanted into nude mice subcutaneously. The weight and size of tumors formed by sh-FGFRL1 cells were significantly decreased in comparison with the tumors formed by the sh-control group (Figure 4(c)). The results showed that FGFRL1 promoted tumor growth in OC.

### 3.7. FGFRL1 Regulates the Hedgehog (Hh) Signaling Pathway

**Figure 5:**
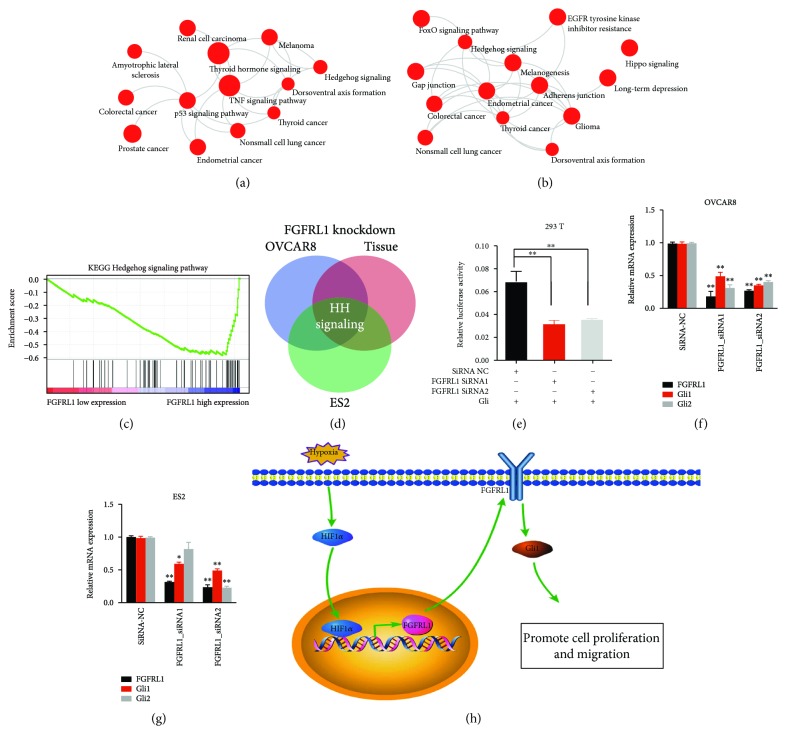
FGFRL1 enhances the Hh signaling pathway. (a, b) Kyoto Encyclopedia of Genes and Genome (KEGG) analysis of cDNA microarray showed gene set enrichment after FGFRL1 knockdown in OVCAR8 and ES2 cells. (c) The GSEA plot based on the gene expression profiles by comparison of the lower FGFRL1 expression group and the higher FGFRL1 expression group in the GSE9891 dataset. (d) The Venn diagram of the significantly altered pathways in two OC cells and ovarian tissues. (e) The activity of the Hh signaling pathway was significantly inhibited after knockdown of FGFRL1 by a luciferase reporter assay. (f, g) The mRNA expression levels of target genes of the Hh signaling pathway in OC cell lines after knockdown of FGFRL1. (h) Schematic summary of the findings presented in this study on the role of FGFRL1 in OC. ^∗^ *p* < 0.05; ^∗∗^ *p* < 0.01.

To elucidate the signaling pathways that were significantly altered following the silencing of FGFRL1 expression, we performed global gene profiling experiments of the OVCAR8 and ES2 cells after knockdown of FGFRL1 using siRNAs. We conducted pathway analysis with the differentially expressed genes in two OC cells. Functional and gene network analysis with differentiated genes revealed significantly altered pathways (Figures 5(a) and 5(b)). Meanwhile, we analyzed a microarray dataset from GSE9891 and divided expression data into two groups named the high expression group (*n* = 19) and low expression group (*n* = 20) according to the expression level of FGFRL1. Gene set enrichment analysis (GSEA) using hallmark gene sets was performed and showed striking alterations in several pathways including the Hh signaling pathway (Figure 5(c)). The cross of the significantly altered pathways in two OC cells and ovarian tissues showed that the Hh signaling pathway was significantly altered after FGFRL1 knockdown (Figure 5(d)).

To further evaluate whether knockdown of FGFRL1 inhibited downstream Hh signaling, the Gli-luciferase reporter was used in luciferase reporter assays. As expected, luciferase reporter assays further confirmed that the activity of the Hh signaling pathway was significantly inhibited after knockdown of FGFRL1 (Figure 5(e)). Furthermore, the mRNA expression level of target genes (Gli1 and Gli2) of Hh signaling in FGFRL1-reduced OC cells was significantly decreased compared to the control, indicating that FGFRL1 silencing inhibited the downstream of the Hh signaling pathway (Figures 5(f) and 5(g)).

## 4. Discussion

In this study, we observed that FGFRL1 was commonly upregulated in both OC cells and tissues compared with normal controls. High FGFRL1 predicted poor prognosis in OC and other tumors. In OC cells, FGFRL1 expression could be induced by hypoxia via HIF1*α*. FGFRL1 exhibited oncogenic functions in promoting cell proliferation and cell migration by activating Hh signaling.

FGFRL1 was demonstrated to express preferentially in skeletal tissues, and small amounts of FGFRL1 mRNA were detected in other tissues such as the heart [2]. In the current study, FGFRL1 was highly expressed in OC tissues and other different neoplastic diseases. However, the reason why FGFRL1 is upregulated in OC is still unclear. Although DNA copy number amplification partly contributed to the increased expression of FGFRL1 in OC, we need more evidence to explain the upregulation of FGFRL1. It has been reported that solid tumor frequently encounters hypoxia stress, especially measurable sized solid tumors and the overexpression of HIF1*α* has been observed in many tumors [24, 25]. In this study, FGFRL1 expression could be induced under hypoxia condition in OC cells, which was consistent with previous study [26]. Our data further demonstrated that HIF1*α* was essential for the hypoxia-induced FGFRL1 expression by transcriptionally binding to the FGFRL1 promoter based on a series of assays.

To evaluate the prognostic value of FGFRL1 in OC, we performed Kaplan-Meier survival analysis. High FGFRL1 expression was associated with poor prognosis in OC patients. This was consistent with the findings in this study that elevated FGFRL1 was an indicator for the poor prognosis in gastric carcinomas.

It was reported that downregulation of FGFRL1 decreased cell proliferation by promoting the proportion of cells in G1/G0 phase and decreasing in S and G2/M phases in human laryngocarcinoma cancer and esophageal squamous cell carcinoma [14, 16]. Our data revealed that no significant difference was observed in cell cycle assay. Herein, we explored that FGFRL1 promoted cell proliferation, inhibited apoptosis and, promoted cell migration of OC cells. The discrepancy might be due to different mechanisms in specific tumors.

FGFRL1 was ever thought to have no effect on ERK1/2 signaling without the intracellular tyrosine kinase domain [7]. However, FGFRL1 was identified to enhance ERK1/2 signaling through association of SHP with the receptor's intracellular SH2-binding motif [12]. Our study demonstrated that Hh signaling was activated by FGFRL1.

In conclusion, we describe FGFRL1 as a crucial factor in the clinical outcome and progression during human OC, indicating it is a novel therapeutic target that can be used for the treatment of OC.

## 5. Conclusions

Our results demonstrated that FGFRL1 was commonly upregulated in OC cells and tissues compared with normal controls. High FGFRL1 predicted poor prognosis in OCs. FGFRL1 expression could be induced by hypoxia via HIF1*α* in OC cells. FGFRL1 significantly promoted cell proliferation and migration of OC cells *in vitro* and tumor growth *in vivo*. FGFRL1 exhibited oncogenic functions in promoting cell proliferation and cell migration by crosstalk with Hh signaling. Taken together, this study provides valuable insight into FGFRL1, which plays an important role in tumor growth and Hh signaling which could serve as potential therapeutic targets for the treatment of OC.

## Supplementary Materials

Supplementary Materials Supplementary Table S1: SiRNAs used in this study. Supplementary Table S2: primers used in this study. Supplementary Table S3: correlations between FGFRL1 expression and clinicopathologic features in OC patients.
