# Supplementary material for: FGFRL1 Promotes Ovarian Cancer Progression by Crosstalk with Hedgehog Signaling
Source: J Immunol Res. 2018 Feb 20;2018:7438608. doi: 10.1155/2018/7438608 (PMC5838460; doi:10.1155/2018/7438608)
Supplement: Supplementary Materials — Supplementary Table S1: SiRNAs used in this study. Supplementary Table S2: primers used in this study. Supplementary Table S3: correlations between FGFRL1 expression and clinicopathologic features in OC patients. [file 7438608.f1.docx]

SUPPLEMENTARY TABLE S1: SiRNAs used in this study.

|  | **Sense (5'-3')** | **Antisense (5'-3')** |
| --- | --- | --- |
| HIF1α_SiRNA1 | GAGGAAGAACUAAAUCCAAdTdT | UGAUACCAACAGUAACCAAdTdT |
| HIF1α_SiRNA2 | GACACCUAGUCCUUCCGAUdTdT | AUCGGAAGGACUAGGUGUCdTdT |
| FGFRL1_SiRNA1 | GCUGGAUGACAUUAGCCCATT | UGGGCUAAUGUCAUCCAGCTT |
| FGFRL1_SiRNA2 | GCCAGAAGUUUGUGGUGCUTT | AGCACCACAAACUUCUGGCTT |

SUPPLEMENTARY TABLE S2: Primers used in this study.

| **Gene** | **Forward sequence (5’-3’)** | **Reverse sequence (5’-3’)** |
| --- | --- | --- |
| FGFRL1 | TGGATGAAGGACGACCAG | TTCTTCAGGCTCAGTGTC |
| Gli1 | AGCGTGAGCCTGAATCTGTG | CAGCATGTACTGGGCTTTGAA |
| Gli2 | CTGCCTCCGAGAAGCAAGAAG | GCATGGAATGGTGGCAAGAG |
| HIF1A | ATCCATGTGACCATGAGGAAATG | TCGGCTAGTTAGGGTACACTTC |
| FGFRL1-1 | CCTATTTTTTTGGTTGCTGCAGCTG | TCAGCCAACCCCATGGGGTGACCGC |
| FGFRL1-2 | GAGGATGGGGCCTGAGACCCCGAGG | TCTGGCGGTGGCGGCGGCGCCGGCG |
| FGFRL1-3 | GAGGGCAGCGCGTGCCGGCGCGGGA | GGCTGCCGGACACAGGTGCGTGGAA |
| FGFRL1-4 | CCCGCACATCTGTGGGAGCGCTGCC | GTCGCGAGGGCGGATGGAGGTGCGC |
| FGFRL1-5 | ACGCCCACGCTGGCCCAGACGCGGG | TCCCGCCTCACACCCTCGACTCCGC |
| FGFRL1-6 | GATGTGGGCAGGCCGTGAGGTTTGT | AGGCCTCCCAGCCTCTCACCTGCTA |
| FGFRL1-7 | CTCAGGTGGGAACATACCTTTGGCC | GACCCCCAGAGCAGGCTCCGGCCCT |
| FGFRL1-8 | TCGCGGAGGTGTCTCGAGAGTTGAG | CGTGCCCCGCCCCGCCGCCAAACTT |
| FGFRL1-9 | CGGGGGAGGGCTGACGCGCGCGGAT | GGCCCGGTCTCGGGGCGGGGCGGCG |
| FGFRL1-10 | CCCGGGGGCGCGGGGCGGCGGGATG | GCGCTGGGGGGGGCCCACGTGGGGC |

SUPPLEMENTARY TABLE S3: Correlations between FGFRL1 expression and clinicopathologic features in OC patients.

|  |  | **Expression of FGFRL1** | | |  |
| --- | --- | --- | --- | --- | --- |
| **Variable** |  | **Low** | **Middle** | **High** | ***P*-value** |
| **Age (years)** | **≤50** | 12 | 27 | 13 | 0.693 |
|  | **>50** | 8 | 28 | 11 |  |
| **Grade** | **G1** | 3 | 10 | 1 | **0.002** |
|  | **G2** | 5 | 14 | 3 |  |
|  | **G3** | 0 | 27 | 18 |  |
| **Clinical stage (FIGO)** | **I** | 4 | 24 | 9 | 0.625 |
|  | **II** | 4 | 7 | 5 |  |
|  | **III, IV** | 2 | 9 | 4 |  |
